# Where London Takes the Air

**Published:** 1924-12

**Authors:** 


### WHERE LONDON TAKES THE AIR.

### THE OZONE OF THE PARKS.

LONDON has the reputation of being the healthiest of great cities. This is due, primarily, to the excellence of its sanitation and water supply. But the wide open spaces of heath and common by which it is encircled and the large area of public parks and gardens within its boundaries proper, undoubtedly play a large part in creating that healthiness. With only a few gaps here and there London is surrounded by a chain of ancient commons, once waste lands, which have been saved for the use of the people, to whom they have belonged from time immemorial, by successive Acts of Parliament. This outer chain is practically continuous from Hampstead Heath and Parliament Hill on the north, with Richmond Park, Kew Gardens, Wimbledon Common, Putney Heath on the south-west, Hainault Forest and Blackheath on south-east, to Epping Forest on the east. Within this chain there are other ancient commons—such as Clapham, Wandsworth and Streatham—and new parks and gardens, the aggregate of which touches close on seven thousand acres, " municipal lungs," which, with the exception of Hyde Park, Kensington Gardens, the Green Park, St. James's Park, Regent's Park, Richmond Park, and Greenwich Park—all royal parks and now maintained by the Crown for the benefit of the public—London owes to the much criticised and despised Victorians.

### Victoria Park.

A century ago the rapid growth of London and the herding together of large sections of the poorer classes were beginning to give concern, both to the philanthropist and the economist. Hygiene, as we understand it now, was virtually unknown, but men like the Prince Consort had the vision to see the physical and moral harm that must ensue when there is no place of recreation in poor and thickly-populated areas. It was at his suggestion that money was found for acquiring and laying out Victoria Park in the East End—the first of all the London municipal parks. A special Act of Parliament was required. It was passed in 1840 and five years later Victoria Park with its 193 acres—it is now 244 acres—was opened to the public.

### " Battersea Fields."

The next lung given to London was Battersea Park and here the task devolved on the Commissioners of Woods and Forests. In 1843 a Royal Commission was sitting to consider means of improving the metropolis and to this Commission Mr. Thomas Cubitt suggested that it would be advisable to lay out the region known as Battersea Fields as a pleasure ground. The Fields had an unenviable reputation. By the river-side they were a marsh; the land beyond had formerly been meadows created in the sixteenth century by the building of a rough embankment. But during the eighteenth century they became a public resort with tea-gardens and public-houses dotted all over them. One of the latter, the Red House, for a time had a vogue as fashionable as that of Vauxhall Gardens. But when Mr. Cubitt made his suggestion to the Commission, Battersea Fields had become a public scandal, especially on Sundays. The pandemonium which had come about there was swept away in 1846 by an Act of Parliament, which empowered the Commissioners of Woods and Forests to " form a royal park in Battersea Fields," and to make an embankment by the Thames. It was a tremendous task as the whole surface of the Fields had to be raised, hundreds of thousands of cubic feet of earth being used chiefly from the Victoria Dock Extension Works.

### Transforming Kennington Common.

The Government decision to transform the hideous mud-flats at Battersea into a beautiful pleasure ground inspired the inhabitants of Kennington to apply to Parliament for the necessary powers to make Kennington Common into a park. The Common, with its memories of the gibbet, was an eyesore, bare of grass the greater portion of the year, and with many ponds and ditches filled with stagnant water. Many difficulties, however, stood in the way. There were some two hundred copyholders who had common rights, and a certain amount of local opposition was encountered on the ground that the people of Kennington would have no place in which to play cricket. Finally, the copyholders were pacified, and to meet the needs of the cricketers the Prince Consort offered the lease of the Oval, which was then a market garden belonging to the Duchy of Cornwall, to any proper authority. This offer led to the formation of the Surrey Cricket Club and the beginning of an important chapter in the history of cricket. All difficulties being removed Parliament passed the necessary Act, the Government undertaking to make the park if the inhabitants would pay the cost, some £1,000, of railing it in.

### From Crown to People.

The maintenance of Victoria, Battersea and Kennington Parks presented a difficulty. At that time there was no central municipal authority in London; consequently the only source of maintenance was the State. Hence they were known as " royal parks." The three parks were placed under the Office of Works and every year Parliament voted the money required for their maintenance, in spite of regular objections from members for provincial constituencies, who protested that the people of London should pay for their own parks. These objections had no effect until 1886, when the House of Commons refused the grant, whereupon the Government, admitting the fairness of the objection, brought in a Bill, which, passing as "The London Parks and Works Act," transferred the charges of these three parks to the then central municipal authority for London—the Metropolitan Board of Works.

THE MISADVENTURES OF FINSBURY.

Finsbury, although its park, in point of history, was the first London municipal park, was not so fortunate as Kennington. All the open spaces, such as the Shepherd and Shepherdess Fields and the Spa and White Conduit Fields, which formerly were available for recreation, had become covered with buildings. The Government lent a sympathetic ear, but before anything could be done it went out of office; and its successor also fell before Parliament had given its authority for the new park. When the question of Finsbury Park was brought before yet a third Government the Act which created the Metropolitan Board of Works was under consideration, and as the Board was to have the power of making parks the Government, not wishing to saddle the public revenue with any further maintenance of open spaces in London, refused to move in the matter. Among the earliest measures obtained from Parliament by the Metropolitan Board of Works was the Finsbury Park Act, 1857. But a bitter disappointment awaited Finsbury. During the previous approaches to Government Lord Palmerston, then Prime Minister, had promised to advise a subsidy of £50,000 towards the cost. When the Act was passed the Government declined to give the assistance, the consequence being that the plan fell into abeyance for many years. In fact it was nearly abandoned altogether, but finally 115 acres were purchased and Finsbury Park was opened in 1869, nineteen years after the project had been set afoot. The delay had a two-fold effect. The original 250 acres chosen for the park on the borders of Finsbury had long ago been built over and the promoters were compelled to buy 115 acres at some distance from Finsbury itself. From this time forward the number of municipal parks in London has increased steadily until now, including the park at Southfields opened last year by the King and Queen, there are about fifty. Besides these there are some four hundred open spaces, such as disused churchyards and burial grounds, and small areas snatched from the devouring hand of the builder, which are the greatest boon in crowded districts. To this list must now be added Ken Wood.

